# Laser Speckle Velocimeter for Mobile Industrial Robot Odometry †

**DOI:** 10.3390/s26196038

**Published:** 2026-09-23

**Authors:** Sam J. Gibson, Thomas O. H. Charrett, Fariborz Eghtedari, Ralph P. Tatam

**Affiliations:** 1 Centre for Engineering Photonics, Cranfield University, Bedford MK43 0AL, UK; sam.j.gibson@cranfield.ac.uk (S.J.G.); r.p.tatam@cranfield.ac.uk (R.P.T.); 2 Manufacturing Technology Centre, Coventry CV7 9JU, UK; fariborz.eghtedari@the-mtc.org

**Keywords:** laser speckle, mobile robot, robot navigation, robot odometry, speckle correlation, robot positioning

## Abstract

This paper reports the design and construction of a laser speckle velocimeter for application to mobile industrial robots. It is intended to supplement the typically used simultaneous localisation and mapping (SLAM) approach by providing enhanced odometry that unlike wheel encoders is unaffected by wheel slippage and will improve navigation in feature-sparse or varying environments. The sensor was installed on an industrial mobile robot (MIR200) and performance trials conducted in a manufacturing environment. We report a novel signal processing scheme that was developed for reliable operation across a range of surface features typically found in these environments, and present the results of odometry benchmarks conducted in a real-world environment. The odometry calculated using the laser speckle sensor was assessed in comparison to the MIR200’s onboard SLAM implementation and to ground truth measurements of robot position using a laser tracker. The overall performance of odometry calculated using the speckle sensor and robot heading was comparable to that achieved by the robot’s SLAM system, showing odometry errors of 0.07–0.46% over a 48 m path. Over the outward path (the first 24 m) the speckle-based odometry performed better than SLAM, with errors of 0.07–0.46% for the speckle and 0.16–1.22% for the robot’s SLAM algorithm. It is therefore anticipated that further performance improvements could be made by fully integrating the sensor output and SLAM data.

## 1. Introduction

Ground-based mobile robots are being used in increasingly varied and challenging environments, such as large construction sites [1,2], underground pipes [2], mines [3], and nuclear facilities [4]. These locations present issues for simultaneous localisation and mapping (SLAM) techniques using LIDAR and depth cameras due to their relatively featureless and/or repetitive environments, in particular long corridors or sewer pipes with parallel walls [1,5,6,7]. Most SLAM approaches incorporate odometry data from wheel encoders to improve their estimations, but these sensors are also susceptible to measurement errors. The two main causes are slipping, when the wheels are skidding on the surface and thus moving more/less than the encoder measures, and crawling, where the robot moves in such a way that the encoders cannot register the movement [8].

Independent velocity measurements that can account for these movements are desirable, and a popular research approach within robotics is the use of optical mouse sensors thanks to their affordability and out-of-the-box functionality. Most computer mice work on the principle of optical flow, taking successive images of a surface at a very high rate (>1000 frames per second) to find the translation of the sensor, and using a small LED (light emitting diode) to illuminate the surface. Some more modern computer mice use laser illumination, adding a laser source to generate a high-contrast interference pattern (speckle pattern) which shifts its position and rotation as the mouse moves across the surface [9]. The pattern is imaged and tracked similarly to the optical flow method. However, since the sensors and their processing are proprietary, it is not always clear which approach a particular device is using in any given study.

Research on the use of mouse sensors for robot velocimetry focuses on optimising them for the different operating conditions, such as surface type and working height. An early study from Palacin et al. [10], looked at the effect on displacement measurements of height changes, diagonal and curved paths, and different floor surfaces. Taking the recommended working height from the manufacturer of 7.45 mm, the paper found that an offset of 0.1 mm in working height led to a 1% systematic error in distance measurements. Diagonal paths also showed an increase in errors, from ∼1% for a straight (1D) path up to 2% for a 45° 2D path, plus a 4% error in the angular measurement. Finally, a test of different surface types found that the systematic errors varied based on surface type: 6.3% on ceramic tile, 5.8% on carpet, 1.2% on stoneware, and 0.8% on wood parquet.

Given these limitations, Bonarini et al. [8] presented results and a calibration method for a robot pose measurement system using two optical mice, mounted on either side of a mobile robot. The calibration involved a manual translation and rotation, used to verify the position and orientation of the mouse sensors relative to the robot chassis, as well as their resolutions. The system was then tested using the UMBmark (University of Michigan benchmark) test for mobile robot odometry [11], in which the robot moves around a 4 × 4 m square (clockwise and anticlockwise) and the difference in absolute and reported pose at the end point are compared for each path direction. The results from Bonarini et al. showed a 4.6% error before calibration, dropping to 0.7% after calibration. A more recent paper by Paijens et al. [12] uses a similar calibration approach for a four-sensor system on multiple surfaces, finding an improvement in positioning errors from 0.28% to 0.16%.

Another group (Hu et al. [13]) using an eight-sensor system opted to compare measurements from all sensors to each other and to the previous measurement set, performing a “consistency check” and allowing anomalous measurements to be discarded reliably. When tested over a path with a transparent sheet to introduce anomalous measurements, the error in the robot pose was 0.65%, compared to an error of over 10% without the consistency check. For a path without the transparent sheet, the positioning error was only 0.28%.

Nagai et al. [9] specifically used a laser computer mouse and speckle pattern tracking, substituting their own 650 nm laser diode. The use of a laser allowed for a much wider range of working heights, due to the higher signal strength, and more consistent results across different surfaces, since this version of the sensor is not reliant on surface features. Their experiments showed position errors of less than 1.6% for heights between 50 mm and 140 mm, and similarly low errors for some heights up to 240 mm. The surfaces tested included black and white paper, aluminium, and carpet, with absolute positioning errors between 0.2–1.6% for the various materials. The same paper also presented results for a much longer outdoor path of 190 m, combining the speckle-based sensor with a gyroscope for improved angle measurements, and finding position errors of less than 0.89% [9].

Considering the issues introduced by changes in working height, many papers have suggested adding a lens with a longer focal length and mounting the sensor higher above the working surface [14,15]. The lens compensates for height variations over a rough or segmented surface, while the greater overall working height reduces the relative effect of these variations. Ross et al. [14] found that at a 50 mm working height with a 32 mm focal length lens, the systematic error for a 1.0 mm height change (i.e., 2.0% height difference) was 0.8%. This is lower than the 1% error quoted above from Palacin et al. [10] for a 0.1 mm change at a working height of 7.45 mm (1.3% height difference).

Furthermore, Ross et al. [14] constructed a complete system with two mouse sensors using their surface quality (SQUAL) value, which gives a measure of the image quality based on its contrast. The SQUAL values were used to weight the results from each sensor, reducing the impact of outliers. They found that the errors over an asphalt surface were 3.04% for a single sensor, and dropped to 1.94% for the weighted pair of sensors. Dahmen and Mallot [15] used an external lens (focal length 4.6 mm) and higher working distance (150 mm) for an eight sensor system. They also incorporated the SQUAL values from the sensors, discarding measurements for which the SQUAL value dropped below a threshold. For a 0.45 m radius circular path and arbitrary 10 m path, they reported positions errors of less than 1.0% across all tests.

Another approach to the height dependency of optical mouse sensors is to use an afocal system, i.e., one with an effectively infinite focal length. Yi et al. [16] developed such a sensor using a pinhole arrangement, using sensor fusion to integrate the output with wheel encoder, gyroscope, and camera measurements. The complete system was tested over a 1.5 × 2.0 m rectangular path on carpet, repeated 20 times, with the trajectory measured first using only the wheel encoders and gyroscope, and then again incorporating the camera measurements (used for orientation) and afocal optical flow sensor. The maximum position error for the wheel encoders and gyroscope only was 7.36%, dropping to 0.62% for the complete sensor with a maximum angular error of 1.0°.

Tajti et al. [17] also used a sensor fusion approach, incorporating two mouse sensors, wheel encoder data, and a three-axes combined accelerometer/gyroscope/magnetometer. They were testing a three-omniwheeled robot, which introduces increased slipping errors due to the alternating contact points on this type of wheel. Their system showed a 1% error over a roughly 0.5 × 0.5 m square path. Another group, Liu et al. [18], used an extended Kalman filter to combine the data from a single mouse sensor, the wheel encoders, and an inertial measurement unit (IMU). The resulting pose errors were less than 0.6% over both simple (1.6 m straight line) and complex (34 m random walk) paths.

Previous work has also investigated the use of custom-built laser speckle velocimetry sensors, rather than using off-the-shelf optical mice components. These approaches integrate high-speed cameras and laser illumination, and allow for much greater control of the signal processing. Aliverdiev et al. [19] report a speckle velocimeter for a robotic skimobile, using stacked rows/columns from speckle images acquired at 400 fps to generate streak patterns and computing the velocity from the streak gradient. Their results show velocity errors of less than 2%. Francis et al. [20] use a similar custom system and report position errors of 0.35% at velocities up to 80 mm/s for simulated motions using a 2D stage system for application to Martian rovers. In this work the 2D normalised cross-correlation was used to directly find the pattern translation between frames recorded at 500 fps. Finally, another custom approach by Jo et al. [21] used two cameras with a central laser, mounted on a robotic arm to simulate robot movement. They measured small movements (10 mm and 10°) in six degrees of freedom, finding position errors of 2.6% for in-plane translations.

The approaches using optical mouse sensors have comfortably achieved better than 1% position errors, tackling the issues these sensors face due to variable working heights by adding long focal length lenses or pinholes. They have also been proven on numerous surface types, including wood, tile, carpet, transparent sheeting, paper, aluminium, and sand. Improvements have also been achieved by applying sensor fusion and combining mouse sensor odometry with data from wheel encoders and gyroscopes. Furthermore, the results from Francis et al. [20] and Jo et al. [21] demonstrate the potential for custom approaches with more control over optical design and signal processing. All of these results are summarised in Table 1.

Although the potential of laser speckle based odometry has been recognised, there has been little reported concerning the positional accuracy achievable over longer distances, or the performance of such sensors in more realistic manufacturing operating environments. At the time of the above studies, these custom approaches were limited in applicability by the volume of image data and transfer speed, as well as CPU processing power available for use on board the robot. The increased availability of low cost, low power and high frame rate cameras, plus the increased performance and improved power usage of CPUs allows a fully customised sensor to now be viable for applications on mobile robots. The motivation for this work is to fill the knowledge gap around the achievable performance and to investigate improvements to signal processing for manufacturing applications.

In this paper we report a novel high-accuracy 2D velocimetry sensor based on laser speckle correlation designed for application to industrial mobile robots. In contrast with existing literature, we report the performance of the sensor in a typical factory floor environment at the Manufacturing Technology Centre (MTC) in Coventry, UK. This custom designed sensor is intended to be used alongside other sensors via sensor fusion, through the standard SLAM approach for mobile robots in manufacturing. It provides data akin to wheel encoder odometry without the errors induced by slipping and crawling, enhancing robot navigation in feature sparse and repetitive environments that cause challenges for SLAM alone [1,5,6,7]. The sensor design is detailed, along with the novel signal processing we have developed and tested for reliable operation across a range of surface features found in manufacturing environments. This builds on our previous work [22] by solving signal processing challenges introduced by low spacial frequency features found on some surfaces. The sensor was tested in situ on a MIR200 industrial robot and compared to the robot’s onboard SLAM algorithm. The presented results include a standard odometry benchmark using a 4 m square (UMBmark test [11]), together with a longer 16 × 8 m rectangular run intended to gauge achievable performance over longer distances. In addition, ground truth measurements of the robot’s position were taken using a Leica Absolute Tracker system. To summarise, the main contributions of our paper are:

- The design and build of a speckle velocimetry sensor for improving odometry performance in mobile industrial robots.
- A custom signal processing algorithm for reliable operation on multiple surface materials and features such as safety tape, rubber tile, and paint.
- Experimental testing and evaluation on a mobile industrial robot (MIR200) in a manufacturing environment, including a long 48 m path, demonstrating successful operation of the velocimetry sensor for odometry.
- Performance benchmarking of the velocimetry sensor and the MIR200’s onboard SLAM against an absolute laser tracker (Leica AT960-XR) for ground truth measurements.

## 2. Materials and Methods

### 2.1. Sensor Design

The speckle velocimetry sensor operates on the principle of speckle correlation, tracking translations in the interferometric speckle pattern formed by the scattering of laser light from an optically rough surface [23]. By cross correlation of consecutive camera frames, the translation of the sensor and therefore robot can be found with high accuracy, and a 2D velocity measurement can be made using the interframe time [20].

The sensor head consists of a laser diode module and CMOS (complementary metal-oxide-semiconductor) camera in a 3D printed housing with a rubber skirt to reduce background light. The laser is a Global Laser Solutions (Abertillery, UK) ACCULASE-PWM-650-5-S (650 nm, 5 mW max output) modulating at 500 Hz and a 20% duty cycle. This modulation serves two purposes: to restrict the overall laser output to class II for laser safety in an industrial environment, and to freeze the speckle pattern during acquisition and reduce speckle blur due to motion. The built-in lens produces an elliptical spot of size 15 × 10 mm, which illuminates the surface at a designed working distance of 120 mm, selected to be compatible with the MIR200 industrial mobile robot (Mobile Industrial Robots, Odense, Denmark) on which the sensor was to be tested.

The camera (XIMEA MQ013RG-ON; XIMEA GmbH, Münster, Germany) also operates at 500 fps, acquiring 512 × 640 pixel images of the illuminated floor surface. In front of the camera is an aspheric lens of focal length 30 mm and pinhole with diameter 5 mm, controlling the magnification (0.33) and speckle size (2–3 pixels), respectively. A diagram and photograph of the sensor head is shown in Figure 1a. A Seeed Odyssey Intel Celeron J4105 mini PC (Seeed Technology Co., Ltd., Shenzhen China), also in Figure 1a, was used for control and data processing, with the built-in Arduino Zero board modulating the laser and synchronising the camera trigger. Figure 1b shows the MIR200 robot with the sensor mounted on the right side of the image.

The above system parameters (imaging lens focal length, camera frame rate and image size, aperture diameter, and laser duty cycle), are all interrelated and selected according to the following constraints.

For a given maximum robot velocity $v_{max}$, the maximum surface translation ($a_{max}$) and maximum speckle shift at the camera sensor ($A_{max}$) are given by:

(1) $$\begin{array}{c} a_{max}=\left|v_{max}\right|\Delta T \end{array}$$

(2) $$\begin{array}{c} A_{max}=a_{max}M \end{array}$$

where ΔT is the interframe time (i.e., inverse of the frame rate), and *M* is the image magnification as determined by the focal length of the lens, *f*, and the sensor working distance. The maximum working speed of the MIR200 robot is 1.1 m/s.

To ensure that the speckle pattern remains correlated before and after translation, the maximum surface translation is constrained by the aperture diameter *d*, to $a_{max}\leq d\;/\;2$ [23]. Additionally, *d* itself should be selected to provide an appropriate speckle size $\langle \sigma_{S}\rangle$ of around 3 pixels, where for a wavelength of λ [20]:

(3) $$\langle \sigma_{S}\rangle =\frac{\lambda f(1+M)}{d}$$

The maximum speckle shift at the camera sensor is constrained by $|A_{max}|\leq N\;/\;2$, where *N* is the correlation image size and the factor 2 is chosen to allow unambiguous interpretation of the shift when using a circular cross-correlation [24]. The correlation image dimension, *N*, is also restricted by the achievable processing rate and camera frame rate. The signal processing described in the following section was developed to increase the $A_{max}$ by increasing *N* whilst maintaining low-cost and high-speed processing.

The frame rate choice and camera pixel size (*p*) will also affect the velocity resolution, δv. For a smallest resolvable shift of 1/20 of a pixel [24]:

(4) $$\delta v=\frac{0.05p}{M\Delta T}$$

Finally, the duty cycle of the illumination is chosen to provide good signal levels in the captured images (see Figure 2a in next section) and also limit the speckle blur, *b*, given by:

(5) $$b=\frac{|v|\Delta tM}{p}$$

Note that Δt is the time the laser is on during its duty cycle, in this case 20% and so Δt=0.2ΔT. The speckle blur has the effect of lowering the correlation peak height, as the peak power is distributed over a larger area. To ensure detection, the peak must remain above the correlation background noise level, which is also dependent on the correlation image dimension *N*. In this work no issues were observed with blur-limited detection.

### 2.2. Signal Processing

The camera runs continuously at 500 fps, with an image size of 512 × 640 pixels. For processing, the images are grouped into batches of ten, with two being used for correlation and the rest discarded. From this the velocimetry sensor can output data at 50 Hz, while giving some flexibility on which frames are used. For this work, only the first two images were used, with a frame spacing of 2 ms, giving a velocity resolution from Equation (4) of δv=0.28 mm/s. In future the remaining frames can be used to provide enhanced velocity resolution at lower speed, by increasing the separation of frames used in the correlation. For the maximum frame separation of ten frames, this would improve the velocity resolution by a factor of ten.

The image processing uses a coarse–fine approach to achieve high-speed and low processing cost 2D correlation. First, the original images (Figure 2a) are binned, reducing the resolution by a factor of eight to 64×80 pixels, as shown in Figure 2b. Then a circular cross-correlation is applied to the binned images, Figure 2c, computed using the Fourier transforms of the two speckle patterns and a single inverse transform [24]:

(6) $$cc\left(u,v\right)=\mathrm{IFFT}(F\left(\xi ,\eta \right)T^{*}\left(\xi ,\eta \right))$$

The result cc(u,v) is the cross-correlation in image coordinates *u* and *v*. F(ξ,η) is the Fourier transform of the first frame, and $T^{*}\left(\xi ,\eta \right)$ is the complex conjugate of the second frame, where ξ and η are the spatial frequency coordinates in inverse pixels (px^−1^).

**Figure 2:**
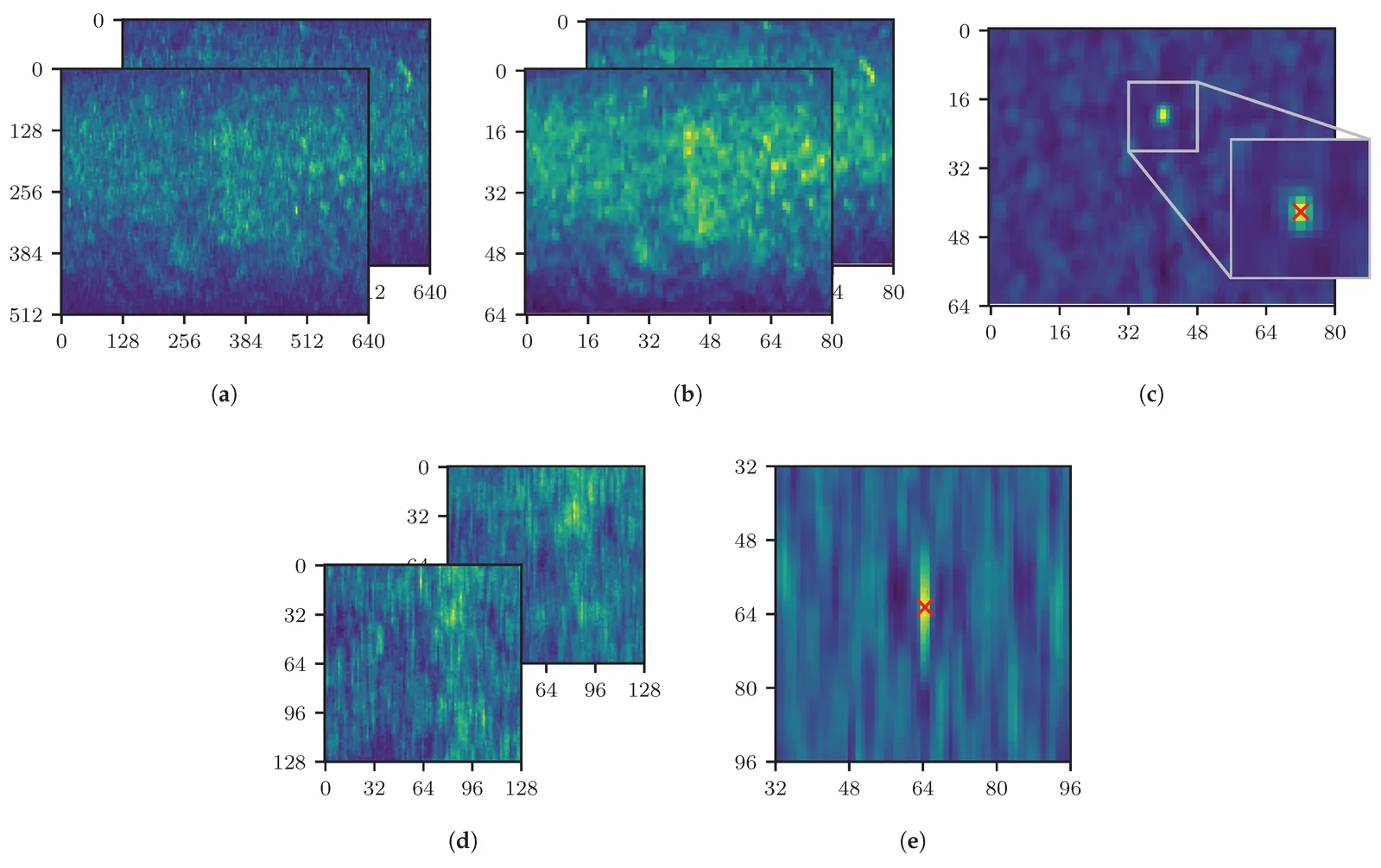
Speckle image processing steps. (**a**) Original pair of speckle images. (**b**) Downsampled speckle images. (**c**) Correlation of binned speckle images, with the peak finding applied. (**d**) Windowed full resolution speckle images. (**e**) Correlation of windowed speckle images, with peak finding applied.

The peak in the correlation image is found using a three-point Gaussian fit in both the x and y directions, giving a rough estimate of the speckle pattern displacement. This rough displacement is used to select two 128 × 128 pixel windows from the original, un-binned images, one from each image. The windows are selected symmetrically around the centre of the image, with their separation as the coarse estimate of pattern displacement. As a result, each window shows the same section of speckle pattern, as can be seen by eye in Figure 2d.

The two windows are correlated using the same circular cross-correlation and three-point Gaussian fit, giving a finer, sub-pixel measure of the speckle shift between the two frames at full sensor resolution (Figure 2e). This is used along with the magnification (0.33) and frame spacing (2 ms) to calculate the instantaneous velocity of the robot.

The coarse–fine approach is much faster and less processing-intensive than correlating the full 512 × 640 image, allowing the sensor to maintain a consistent 50 Hz output and to operate on low-power hardware. The choice of window size is selected to balance correlation noise, which decreases with increasing image size and thus speckle count, and processing load.

### 2.3. Filtered Signal Processing

Testing on multiple surfaces uncovered an issue with images containing low spatial frequency features that correspond to physical surface details rather than the laser speckle pattern that we are tracking. Although such features can be tracked and will give a velocity estimate in a similar manner to our sensor, their scale and nature often leads to the so-called barber’s pole illusion [25], where the apparent motion can be different from the actual motion due to aperture effects. In correlation terms this has the effect of smearing the correlation peak, leading to an ambiguity in the translation direction, and/or false peak detection.

For example, Figure 3a shows a speckle image taken when driving over striped black and yellow warning tape. The sharp line between the high and low intensities correlates with itself along the full length of the line, causing a broad correlation feature that obscures the real peak from the speckle pattern correlation (Figure 3b). It should be noted that although the signal level from the black portion of the tape is significantly lower than that of the yellow portion, speckles are still observed in this region and will contribute to the correlation peak, as shown in the inset.

To overcome the issues with low spatial frequency features and reliably recover the true peak from the laser speckle information only, an intermediate filter stage is added to the computation of the circular cross-correlation:

(7) $$\begin{array}{cc} \; & fcc(u,v)=\mathrm{IFFT}(C(\xi ,\eta ))\\ \; & C\left(\xi ,\eta \right)=\left\{\begin{array}{cc} M\left(\xi ,\eta \right)F\left(\xi ,\eta \right)T^{*}\left(\xi ,\eta \right) & M(\xi ,\eta )>0\\ 0 & M(\xi ,\eta )\leq 0 \end{array}\right. \end{array}$$

Here, fcc(u,v) is the filter correlation result, and M(ξ,η) is the multiplicative mask containing the filter. As the FFT is already calculated as part of the circular correlation (6), this imposes no additional processing load and can in fact reduce computational cost as masked values are excluded from the multiplication.

The applied filter is circular around the origin, with the low-cut section calibrated to remove the lower frequencies relating to surface intensity variations. A high-cut filter is also applied, using the upper limit of the speckle field’s spatial frequency [23], thus removing any high-frequency noise without compromising the speckle information. The filter values are given in terms of the spatial frequency in px^−1^, with a maximum absolute value of 0.5 px^−1^.

For the work presented here, the lower and upper values of the filters were chosen as 0.05 px^−1^ and 0.45 px^−1^ for the binned correlations, and 0.07 px^−1^ and 0.45 px^−1^ for the windowed correlations. These values were chosen as they allowed successful identification of the speckle correlation peak even in the presence of low spatial frequency surface details. The values were found to be suitable for all surfaces tested, as described below in the results section. Figure 4a shows the filter for the windowed correlation, with Figure 4b showing the resulting filtered windowed correlation for the image of the black and yellow warning tape in Figure 3a.

### 2.4. Experimental Trials

Experimental trials were carried out at the Manufacturing Technology Centre (MTC) in Coventry, UK. The environment is typical of an industrial warehouse or factory, with a sealed concrete floor plus features such as expansion gaps, warning tape, paint, and oil.

The sensor was mounted on a MIR200 industrial mobile robot, shown in Figure 1b, which is designed for small–medium logistics tasks. The robot uses a SLAM algorithm with a quoted positioning accuracy of ±50 mm. The position, speed, and heading data from this algorithm was recorded from the robot at 10 Hz, via the robots REST (representational state transfer) API (application programming interface) and wireless network. At this stage no direct integration of the speckle velocimetry sensor data with the internal MIR navigation stack was attempted; instead, odometry was calculated by combining the recorded speckle velocimetry data and the heading provided by the MIR robot.

The odometry data from the MIR’s SLAM algorithm and the speckle velocimetry sensor were both compared to ground truth measurements from a Leica Absolute Tracker AT960-XR (Leica Geosystems, Heerbrugg, Switzerland). This laser tracker has a quoted performance of 14 μm for ranges under 10 m. Throughout each experiment run, the MIR’s position was tracked continuously by the Leica system using a retroreflector mounted on top of the robot.

## 3. Results and Discussion

### 3.1. Velocimetry Measurements on Different Surfaces

Using the improvements described above, the image processing was tested on datasets recorded with short movements across a number of different surfaces typical to industrial environments. These included the aforementioned black and yellow warning tape, other types of tape, oil and paint marks, and rubber tile.

Figure 5 shows a photo of each surface and a representative speckle image including its speckle contrast, plus a graph with the measured velocity and the *q* values for the peak fitting during the fine correlation. The speckle contrast is calculated from the filtered speckle image used during signal processing, which removes the spacial frequency components associated with surface material features whilst retaining the speckle pattern. Since this results in an image with a mean of zero, the images intensities were shifted so that all were positive, and then the contrast could be calculated in the standard way [23]. The *q* values are a ratio of the correlation peak height over the average absolute value of the background noise, i.e., the signal-to-noise. A value greater than 2 indicates that the correlation peak is above the noise level and can be located with the Gaussian fit [20].

Overall, the results are demonstrative of the true robot motion, showing a sharp acceleration, smoothly varying velocity, and sharp deceleration. Each dataset shows a continuously varying velocity from the speckle sensor, with no anomalies or dropouts. Note that the robot was controlled manually for these tests, hence the variation in speed and acceleration. The velocity results for the rubber tile show four small jumps, which appear to coincide with the robot’s wheels crossing the lip of the 6 mm tile (two on, two off). The *q* values and continuous velocity measurement both indicate this is a real effect reflected in the data, and not a signal processing failure.

Despite the apparent variation in contrast in the images themselves, the speckle contrasts are reasonably similar across the nine surfaces, ranging from 0.12 to 0.25. Our analysis of the data shows no discernible relationship between material speckle contrast and correlation *q* value.

However, there is a relationship between q value and robot velocity, and hence speckle blur. When the robot and sensor are stationary, the *q* value stays roughly constant at between 70 and 80. As the robot accelerates, the *q* values drop to between 20 and 50 depending on surface and speed, but for all tests remained well above the minimum threshold of 2. For example, the data for the white spray paint in Figure 5e shows the robot continuing to accelerate as it moves across the surface, with a velocity change from around 0.1 m/s to 0.2 m/s and a corresponding change in *q* values from about 40 to around 30. To further investigate this trend, Figure 6 shows the data from all nine surface material drives plotted as q value against the absolute velocity. The black markers are for the drive over the white paint only, which has the greatest velocity change and shows that the results for each surface are not clustered around one particular point. The data for all runs shows a cluster of points at high *q* values when the robot is stationary or stopping, with a general trend towards lower *q* values at higher speeds. The data is too scattered to conclusively state the shape of the trendline, and further research would benefit from testing a wider variety of robot velocities.

### 3.2. Odometry Benchmark Testing: UMBmark, 4 × 4 m Path

In order to better compare the results of our purpose built sensor with previous work, the first trial followed the procedure of the UMBmark standard odometry test [11]. This consists of the robot driving around a 4 × 4 m square both clockwise (CW) and anticlockwise (ACW) multiple times. Figure 7 shows one complete clockwise traversal of the square, with position data for the speckle odometry sensor (recorded at 50 Hz), the MIR’s built-in SLAM (10 Hz), and the Leica laser tracker (30 Hz). All data have been converted to the laser tracker’s frame of reference for consistency and comparison.

For this run, the speckle odometry is much closer to the ground truth position from the laser tracker at the end of the square than the MIR’s SLAM data, as seen in Figure 7a. The absolute magnitude of the error in the speckle odometry is 4.8 mm, compared to 16.1 mm for the MIR robot. However, this is not indicative of the whole path, as inset Figure 7c shows a section where both the SLAM and the speckle odometry positions are similarly far from the laser tracker position. Also of note is the point at Figure 7b, where the SLAM updates its location estimate leading to a sudden jump in measured position.

The UMBmark test compares the absolute and reported end positions of the robot, checking for overall accuracy and for any systematic errors between the clockwise and anticlockwise paths. Figure 8 shows this data for 12 total runs (6 clockwise, 6 anticlockwise), for both the onboard SLAM and the speckle odometry. The Leica laser tracker data is used for the ground truth end position of each run. The circled markers show the averages for the clockwise and anticlockwise runs, as outlined by the UMBmark procedure.

Considering the average offset, the accuracy for both sensors is very similar; the absolute magnitudes of the offsets for the MIR’s SLAM data are 8.8 mm for clockwise and 14.9 mm for anticlockwise, and the corresponding errors for the speckle data are 5.1 mm and 13.1 mm. As a percentage of distance travelled, these are equivalent to 0.05% and 0.09% for the MIR, and 0.03% and 0.08% for the speckle odometry. This result for the speckle sensor gives an order of magnitude improvement compared to the literature on odometry sensors using optical mice and other custom speckle-based approaches.

From Figure 8, the MIR data show greater variation for the anticlockwise paths, while the speckle odometry has very similar variation for both directions. We believe this is due to the particular features surrounding the 4 × 4 m path: three sides of the square had distinct features such as workbenches and tool chests, whereas the fourth was a relatively featureless corridor. For the anticlockwise paths, this corridor formed the first side of the square, giving the SLAM fewer features to track initially. Since the speckle odometry tracks its position using the floor, it was unaffected by this difference in surrounding features.

We can additionally assess the sensor performance at each corner of the 4 × 4 m square; Figure 9 shows the position errors at each corner, taken from before the robot’s 90° rotation. As indicated by the UMBmark analysis, the MIR position errors have more variation during the anticlockwise runs compared to the clockwise runs, with the variation increasing with distance travelled. For the clockwise data, there is no clear trend of increasing or decreasing errors with distance travelled.

On the other hand, the positioning accuracy of the speckle odometry after the first 4 m is significantly better than that of the MIR robot’s SLAM. The magnitude and variation of the position errors increase with distance, which is expected since the technique measures the position change cumulatively, and so any errors in measurement will also be cumulative. Despite this, the position errors at the end of path, at 16 m, are broadly similar in variation and absolute value to the errors in the MIR/SLAM data.

It should also be noted that the speckle odometry used here is currently completely dependent upon the MIR’s SLAM algorithm estimate of orientation. As such, this may be a factor in the speckle odometry’s position errors, especially for anticlockwise runs and after the first robot rotation. A fully integrated sensor including fusion with inertial measurement units (IMUs) or other independent heading measurements may be expected to perform better.

### 3.3. Performance Assessment over Longer Paths: 16 × 8 m Path

We also conducted experiments using a much longer 16 × 8 m path, to investigate sensor performance for a greater distance travelled. This trial was conducted in the relatively feature-sparse environment of an empty manufacturing hall and without a predetermined SLAM map. The MIR robot was driven clockwise around a rectangular 16 × 8 m path, pausing every 4 m to provide a reference position. Again, laser tracker data was simultaneously acquired to provide a ground truth reference.

We have two complete traversals of this path, plus partial data from a third which will be discussed in more detail below. One of the complete runs is shown in Figure 10. As in the previous trial, all data has been converted to the laser tracker frame of reference, but in this case the retroreflector was offset to the side of the robot, leading to the 90° arcs at each corner.

The MIR and speckle data both align very closely with the laser tracker data for the first side of the rectangle, with the measured *y* position staying within ±35 mm of the laser tracker data for the MIR data, and within ±20 mm for the speckle odometry. At 16 m travel before the first rotation, shown in inset Figure 10b, the speckle odometry is closer to the laser tracker position, with an absolute error (i.e., magnitude of the error vector) of 17.1 mm, compared to 41.4 mm for the MIR’s SLAM. Figure 10c is representative of the position errors for both the MIR and the speckle odometry along the 24–40 m leg of the path, with the offset in *y* for the speckle sensor staying within 5–50 mm, and within −85–−45 mm for the MIR robot. When the robot returns to the start/end point, shown in inset Figure 10a, both datasets have a similar absolute position error as compared to the laser tracker information: 65.0 mm for the speckle, and 69.4 mm for the MIR.

This can also be seen in Figure 11, which shows the position errors for the stationary points at every 4 m. It is difficult to draw out a clear trend due to the small number of data points, but the position errors generally increase as the distance travelled increases. Figure 12a shows the same data but as the absolute magnitude of the error vector. This graph shows more clearly that the speckle odometry errors increase with distance travelled, as expected due to the additive nature of the odometry calculation. However considering these errors as a percentage of distance travelled, shown in Figure 12b, the speckle odometry errors are relatively consistent across the full 48 m, with a minimum and maximum of 0.07% and 0.46% between the two runs, and an average across all measurements of 0.16%.

A trend in the SLAM data is much less clear, roughly showing an increase and then decrease in the absolute position errors as the robot traverses the path. This agrees with expected results, since SLAM will use the mapping data acquired when the robot was at the start position to better estimate its location when it returns to the same spot. The percentage errors for the SLAM show a sharp drop-off after the initial measurement at 4 m, though they continue to show more variation than the speckle odometry errors. The average percentage error for both runs across the whole path in the SLAM data is 0.31%, with a range of 0.07–1.22%.

Comparing the two, the speckle odometry consistently outperforms the SLAM for the first 16 m leg of the path (before the first turn), and performs similarly or better for the remainder. To reiterate from above, the speckle odometry is reliant on the SLAM’s heading output, so errors from the orientation measurement are impossible to separate from the errors in the speckle velocimetry. However, we can note that the highest errors for the speckle odometry are smaller than the highest errors for the SLAM.

Even across this larger path, the position errors in the speckle odometry data are similar or better than those reported for sensors derived from optical mice (see Table 1). In many cases, our sensor also has the advantage of fewer components and/or a smaller footprint; for example, Paijens et al. [12] reported the same positioning error of 0.16% but used four mouse sensors. We have also improved on the existing work on custom speckle-based velocimetry/odometry sensors, with our sensor achieving lower position errors than the 0.4% from Francis et al. [20] and 2.6% from Jo et al. [21].

In addition, the 16 × 8 m path and sealed concrete floor are much more representative of a manufacturing environment than previous work. All but one of the papers summarised here use path lengths of less than 16 m, and trials are mostly conducted on outdoor surfaces [9,14,20], carpet [8,16] or paper [18,21] (again see Table 1).

The consistent performance of the speckle odometry across the larger distances demonstrates its suitability for manufacturing applications. We also suggest that data from a speckle odometry sensor could be incorporated into a mobile robot’s SLAM algorithm to improve its positioning estimates, which is supported by the reported results on sensor fusion with optical mice sensors [16,17,18].

### 3.4. Signal Processing Challenges and Further Work

As mentioned above, a third run of the 16 × 8 m path was attempted but provided limited data due to debris being picked up by the sensor. Figure 13a shows one of the speckle images with this debris (likely hair or dust), alongside the resulting binned 8× downsampled correlation in Figure 13b. The correlation image has two peaks: one in the centre indicating near-zero velocity resulting from the debris, and an off-centre peak from the speckle pattern correlation at the true robot velocity. Since the central peak has a greater intensity, this is the one located by the peak fitting algorithm, leading to a failed correlation.

Tuning of the filtering scheme alone was unable to reliably select for the correct peak, but we believe this could be achieved in the future with an improved peak detection method. To demonstrate this point, Figure 13c shows a successful windowed correlation using manually-selected 128 × 128 pixel windows separated by the speckle shift estimated by eye from Figure 13b. Additionally, omission of the rubber skirt around the sensor (used for these trials as additional eye protection) would likely reduce or prevent the pickup of debris.

## 4. Conclusions

We have presented a complete speckle odometry sensor based on the principle of laser speckle pattern correlation, and demonstrated its application in a realistic manufacturing environment through trials on an industrial mobile robot. We developed a coarse–fine image correlation processing approach and also describe a low computational cost filtering applied to the speckle images before correlation. This filter removes low-frequency noise associated with large scale surface features, such as striped warning tape, that can lead to erroneous peak detection and hence velocity errors.

Trials were carried out at the Manufacturing Technology Centre (MTC), Coventry, UK, applying the sensor to a MIR200 industrial robot. We tested various surface features common to manufacturing environments such as expansion gaps, oil spills, and safety tape. The filters used were tuned to ensure that the speckle sensor performed well for all features, and reliable operation was found to be possible for all surfaces tested.

Further trials looked at the sensor performance over square and rectangular paths, comparing reported positions to ground truth data from a Leica laser tracker. Results from the UMBmark test showed very similar performance for the MIR’s onboard SLAM and the speckle odometry, with the SLAM achieving average position errors of 0.05–0.09% and the speckle achieving 0.03–0.08%. Notably, the speckle sensor performed more consistently than the SLAM on runs where the first stretch of path had fewer surrounding features.

A longer rectangular path of 16 × 8 m showed the expected increase in position errors with increased distance for the speckle odometry sensor. However, the errors as a percentage of distance travelled remained relatively similar over the whole path, ranging from 0.07 to 0.46% and averaging 0.16%. The position errors for the MIR robot’s SLAM had a broader range of 0.07–1.22%, and an average of 0.31%. These results confirm that the speckle odometry sensor could provide complementary data for sensor fusion by a SLAM algorithm for navigation.

We have achieved similar performance to previous work using multiple sensors [12] with average position errors of 0.16% or less, and improved on results for single sensors whose errors range from 0.2 to 1.9% [9,14,16,18]. Furthermore, our results were obtained over a long path in a realistic application environment, demonstrating the benefits of a custom design and signal processing chain. This full control over the image processing has allowed us to identify and solve failure modes such as issues caused by low spatial frequency intensity variations, as with the black and yellow safety tape, without relying on redundancy and multiple sensors. Finally, we have identified a future direction of research in improving our peak detection algorithm to reduce failure modes from trapped debris beneath the sensor.

## Figures and Tables

**Figure 1:**
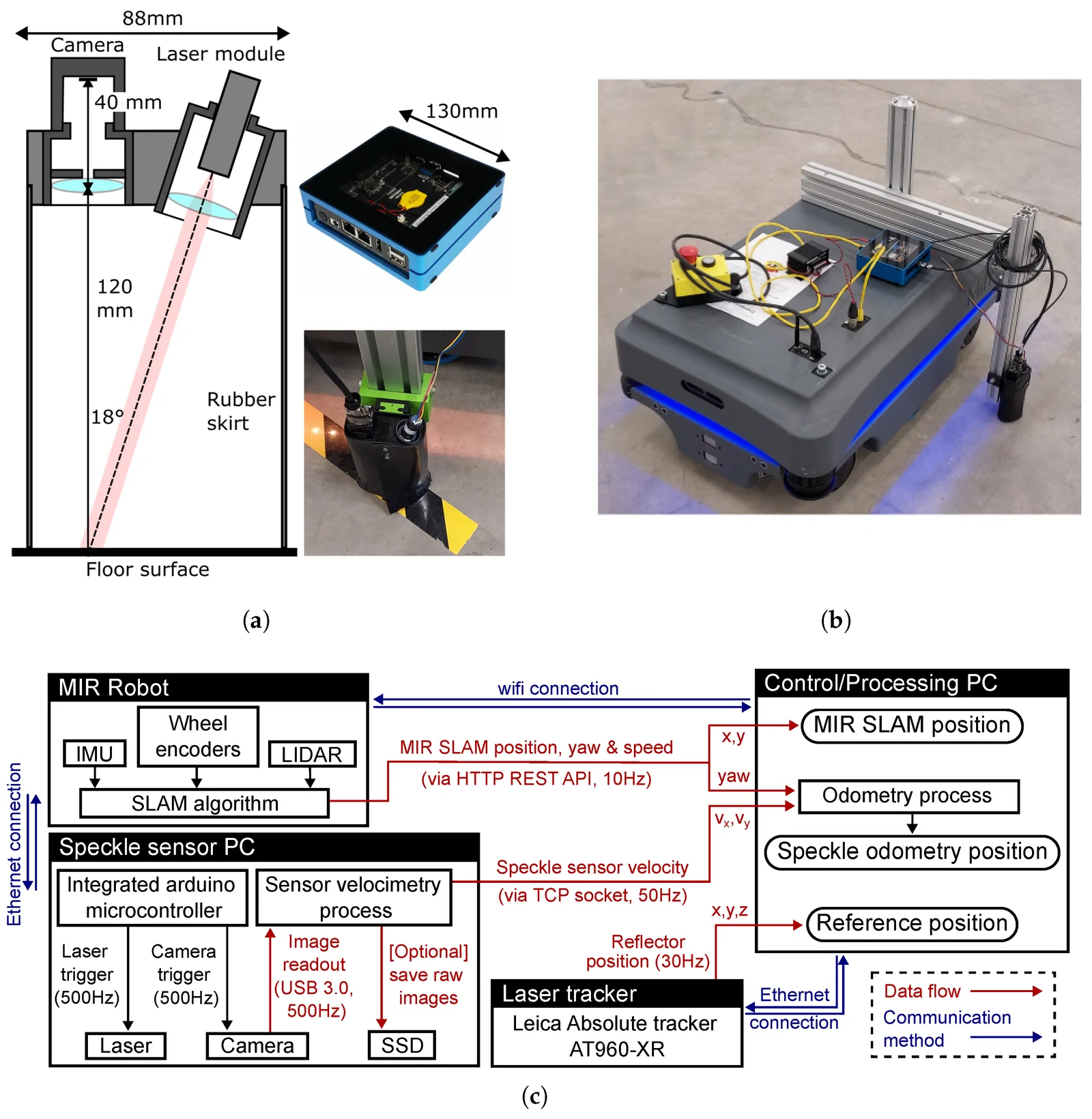
(**a**) Diagram of the speckle velocimetry sensor head (**left**), along with a photo of the mini PC used for processing (**top right**) and a photo of the sensor head in situ (**bottom right**). (**b**) MIR200 industrial mobile robot used for trials, with the speckle velocimetry sensor mounted on the rear left of the robot (robot is facing bottom left) and the mini PC on top. (**c**) Block diagram of data transfer and communication method between the various sensors, sensor components, and PCs.

**Figure 3:**
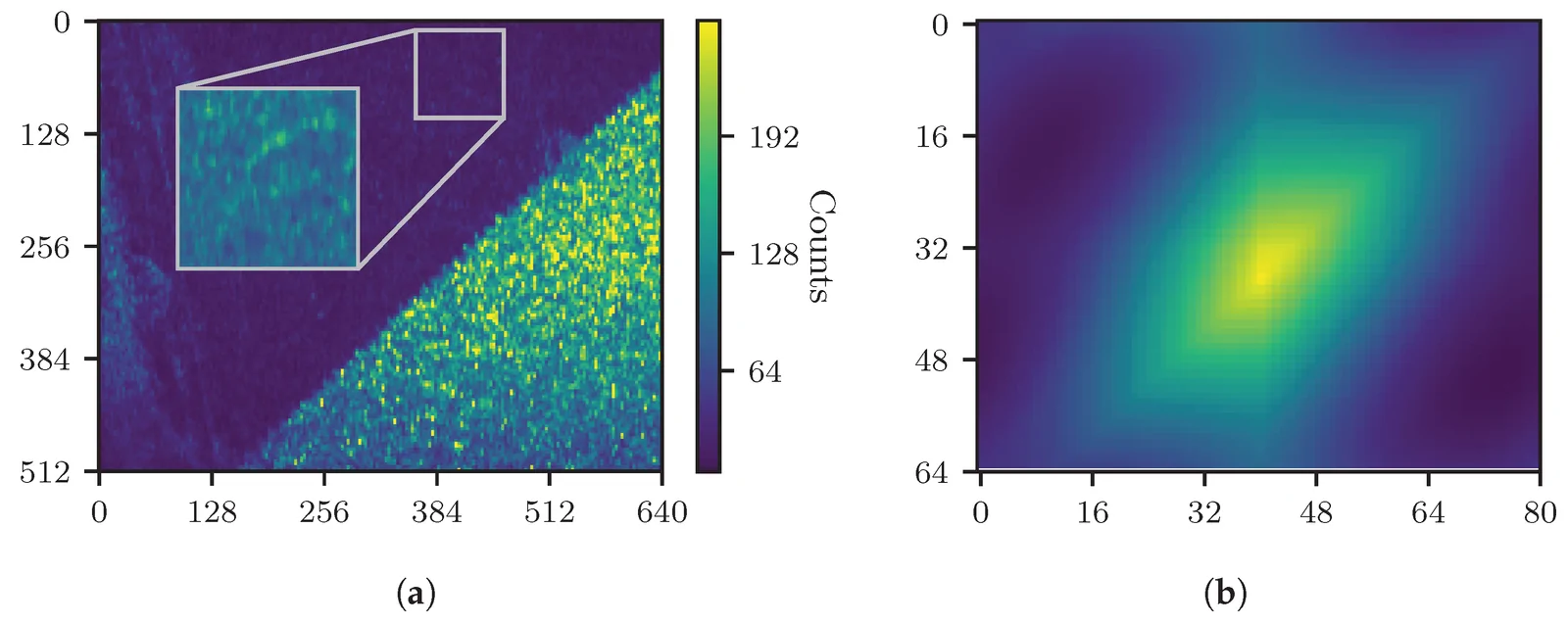
(**a**) Speckle image from black and yellow safety tape. The inset has a restricted colour scale, showing the lower intensity speckle pattern from the black portion of the tape. (**b**) Binned correlation image with broad correlation feature obscuring peak.

**Figure 4:**
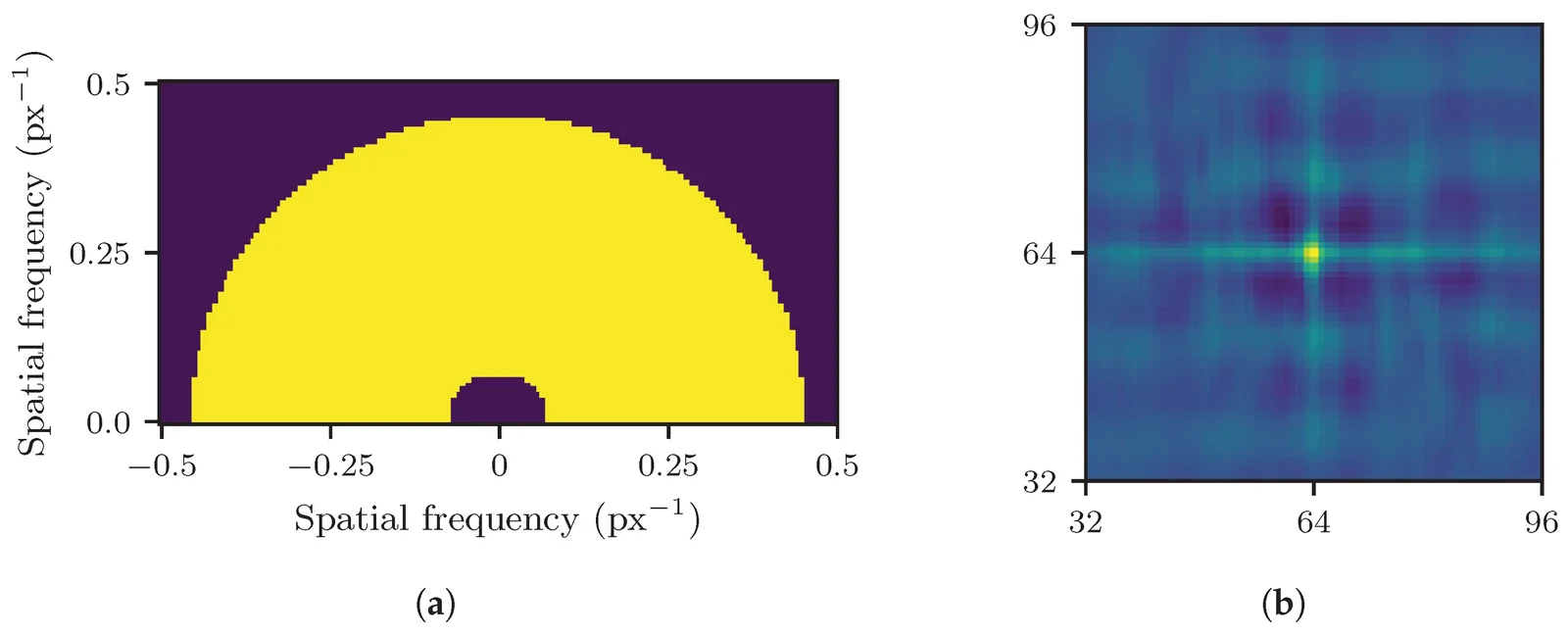
(**a**) Filter applied to speckle image FFT during windowed correlation. (**b**) Filtered windowed correlation for the speckle image in Figure 3a, clearly showing the speckle peak in the centre.

**Figure 5:**
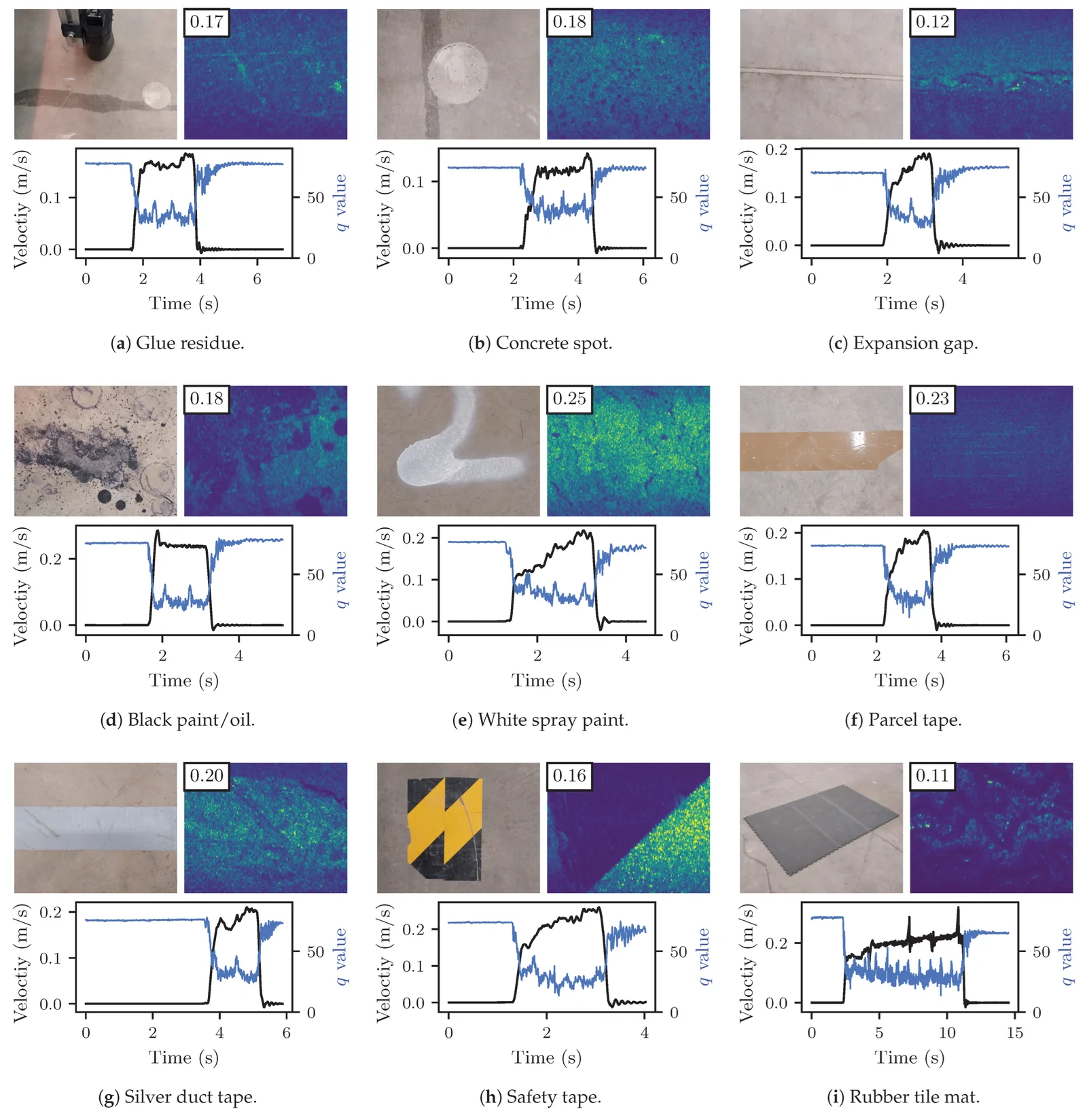
Photograph, speckle image including speckle contrast (value top left), and graph with velocity measurement (black line) with *q* values (blue line) for a short drive over various surfaces. Note that the speckle images in (**a**,**c**,**d**,**f**) have a restricted colour scale to more clearly show the speckle pattern.

**Figure 6:**
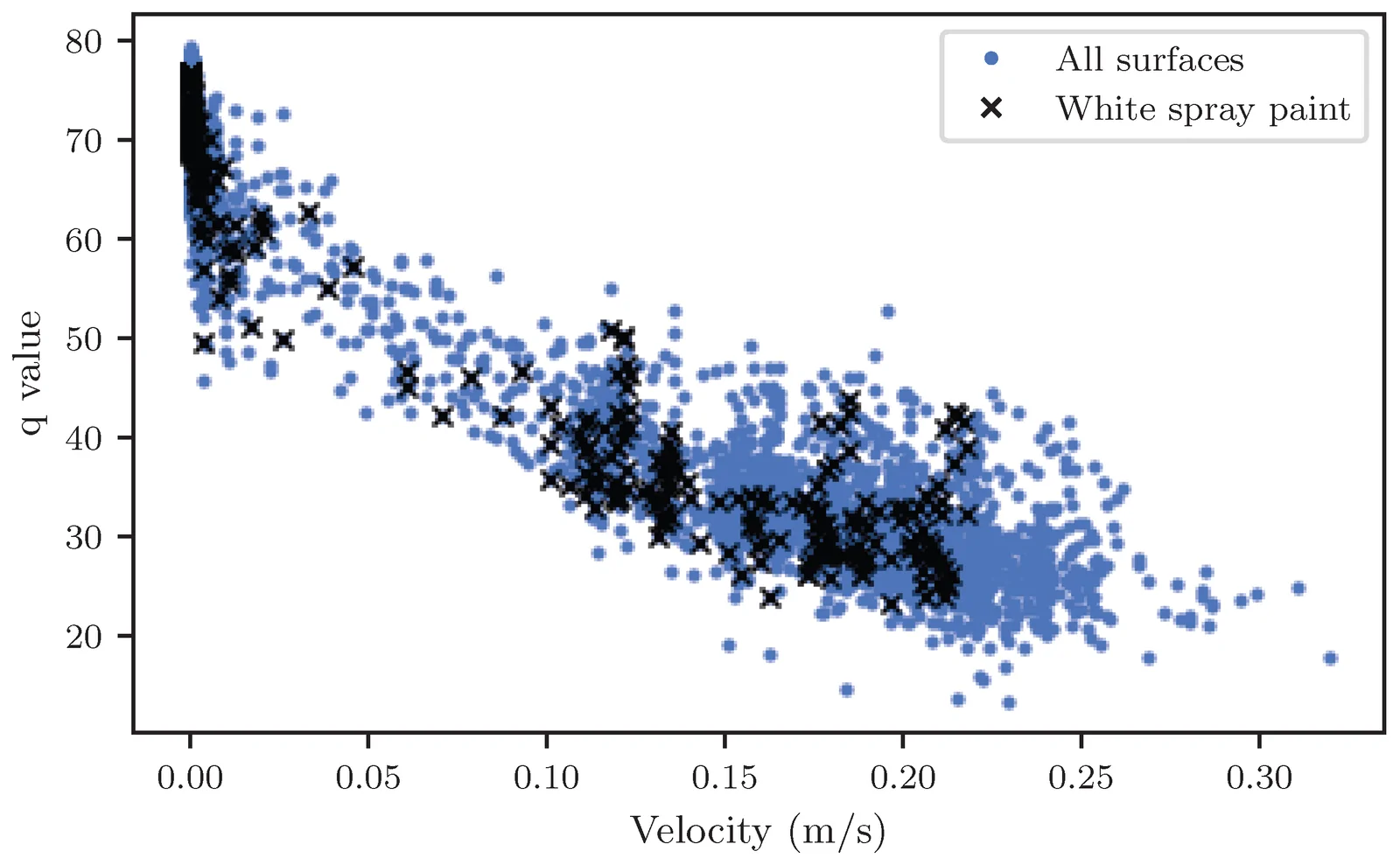
Scatter plot of all *q* values against velocities for the nine surfaces tested (blue markers), with the single run for the white spray paint overlaid (black markers).

**Figure 7:**
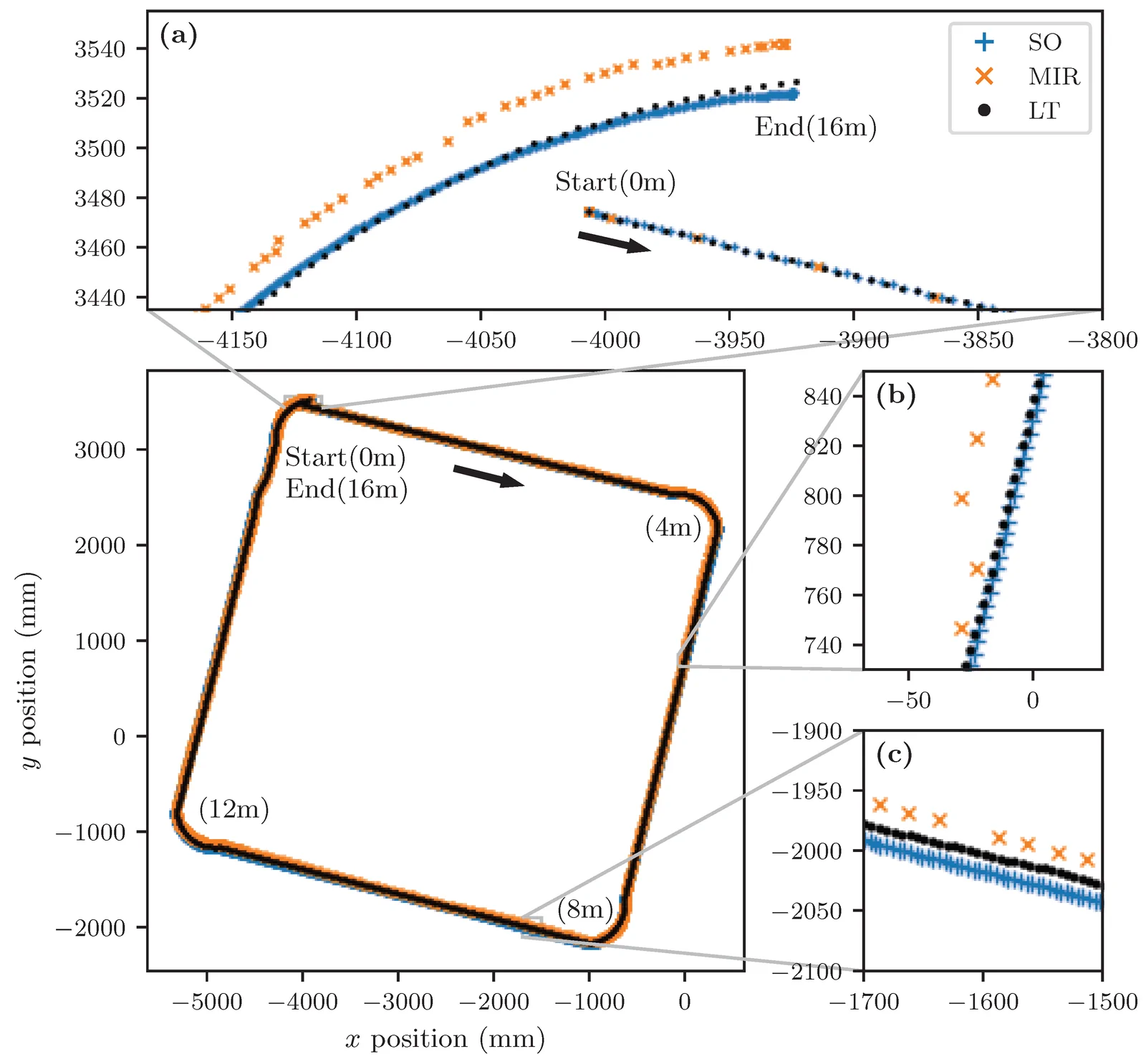
Clockwise run of the 4×4 m square path used for UMBmark calculations, showing odometry from the speckle sensor (SO), the MIR robot’s SLAM (MIR), and position data from the laser tracker (LT). All data are for the robot centre converted to the reference frame of the laser tracker. Inset (**a**) shows the start and end positions; inset (**b**) shows a point where the SLAM updated its position estimate; and inset (**c**) shows an average section along the third leg of the path.

**Figure 8:**
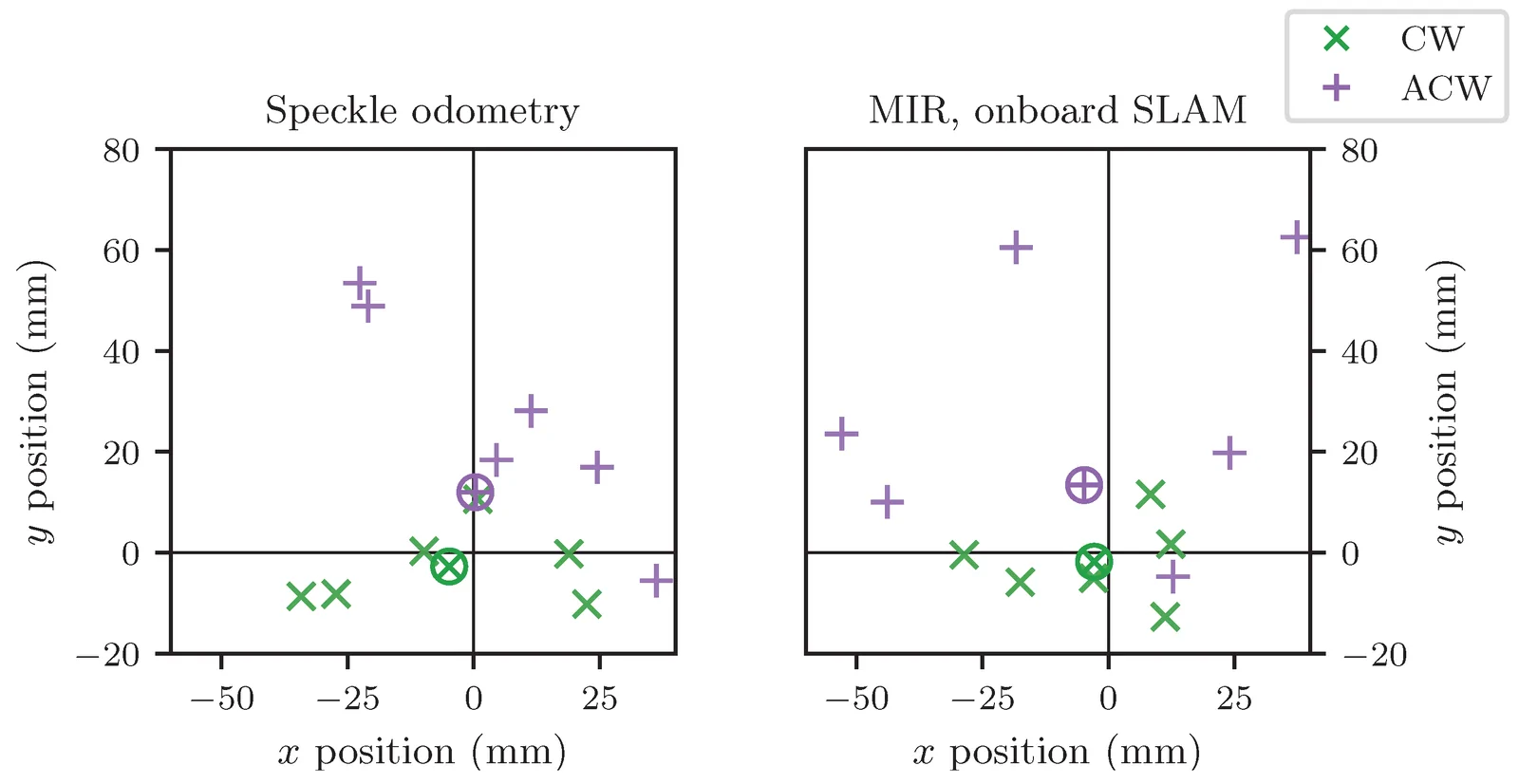
Results for the UMBmark test for 12 traversals of a 4 × 4 m square, six clockwise (CW) and six anticlockwise (ACW); the average offset is given by the circled marker.

**Figure 9:**
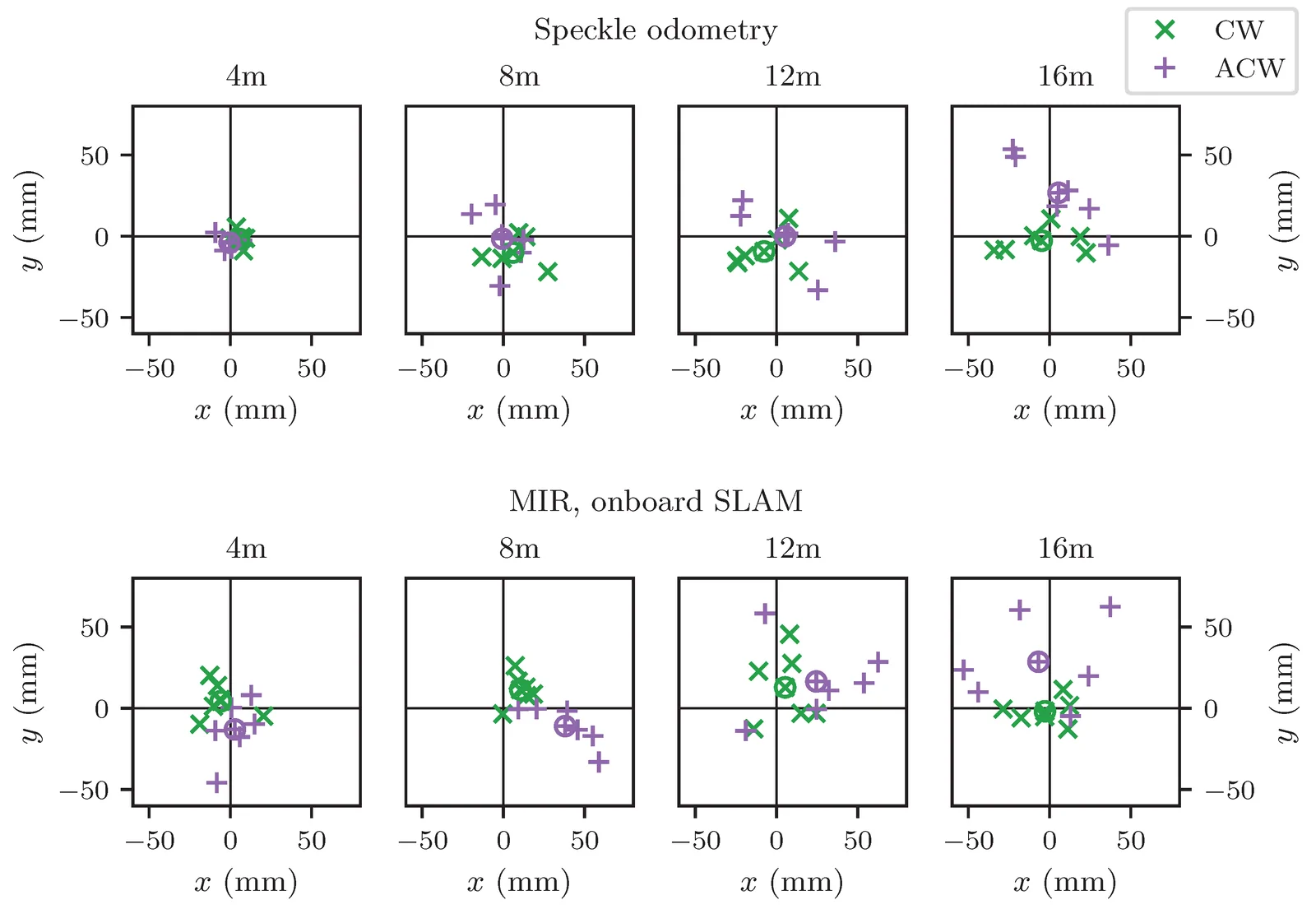
Position errors calculated from the laser tracker data at the four corners of the 4 × 4 m path, for all 12 runs (6 CW, 6 ACW); the average offset for each distance is shown by the circled marker.

**Figure 10:**
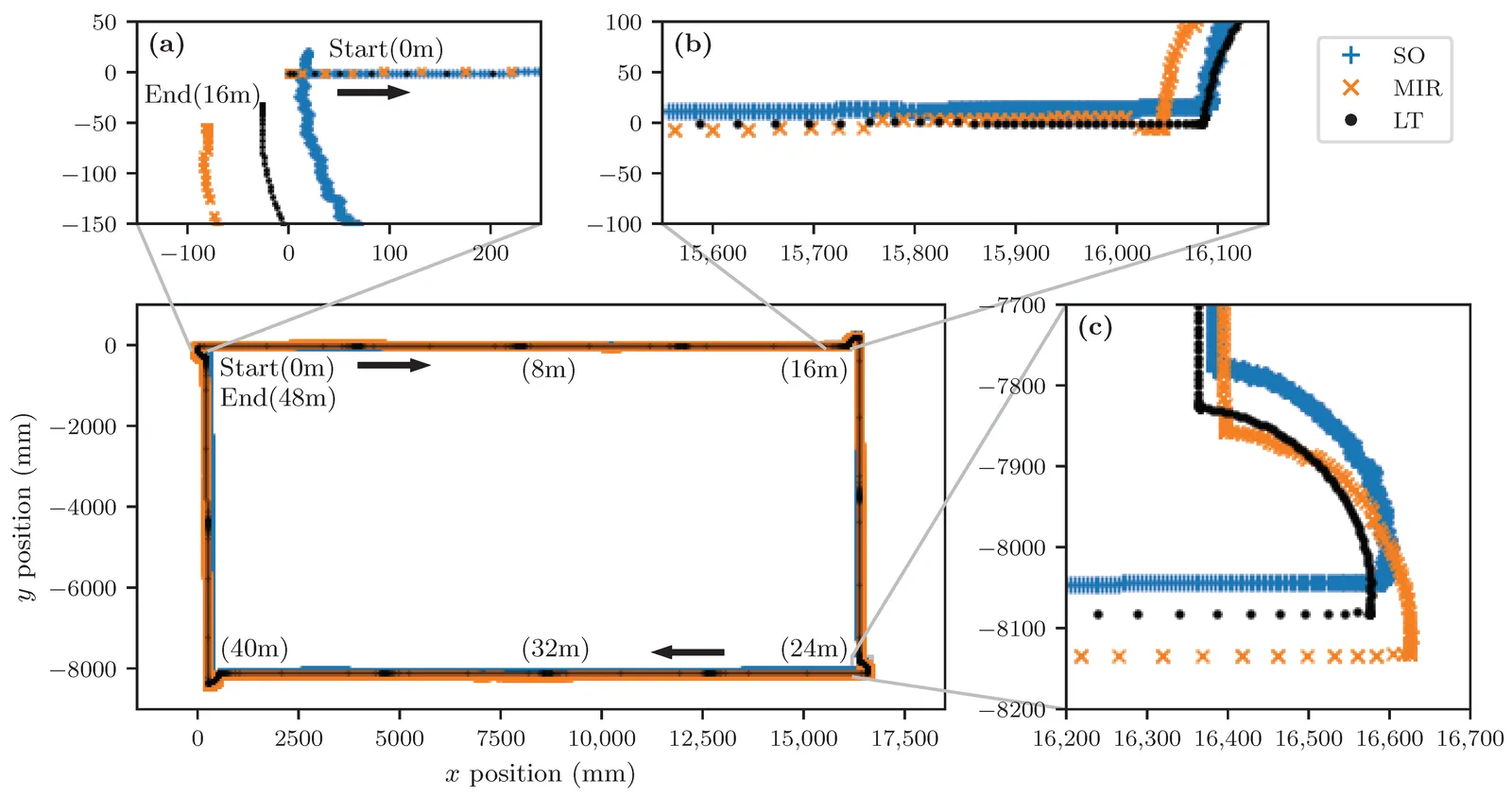
Single run of a 16×8 m path, showing odometry from the speckle sensor (SO), the MIR robot’s SLAM (MIR), and position data from the laser tracker (LT). All measurements have been converted to the laser tracker reference frame, looking at the position of the laser tracker reference point. Inset (**a**) shows the start and end positions; inset (**b**) shows the approach to the first 90° turn; and inset (**c**) shows a full 90° rotation.

**Figure 11:**
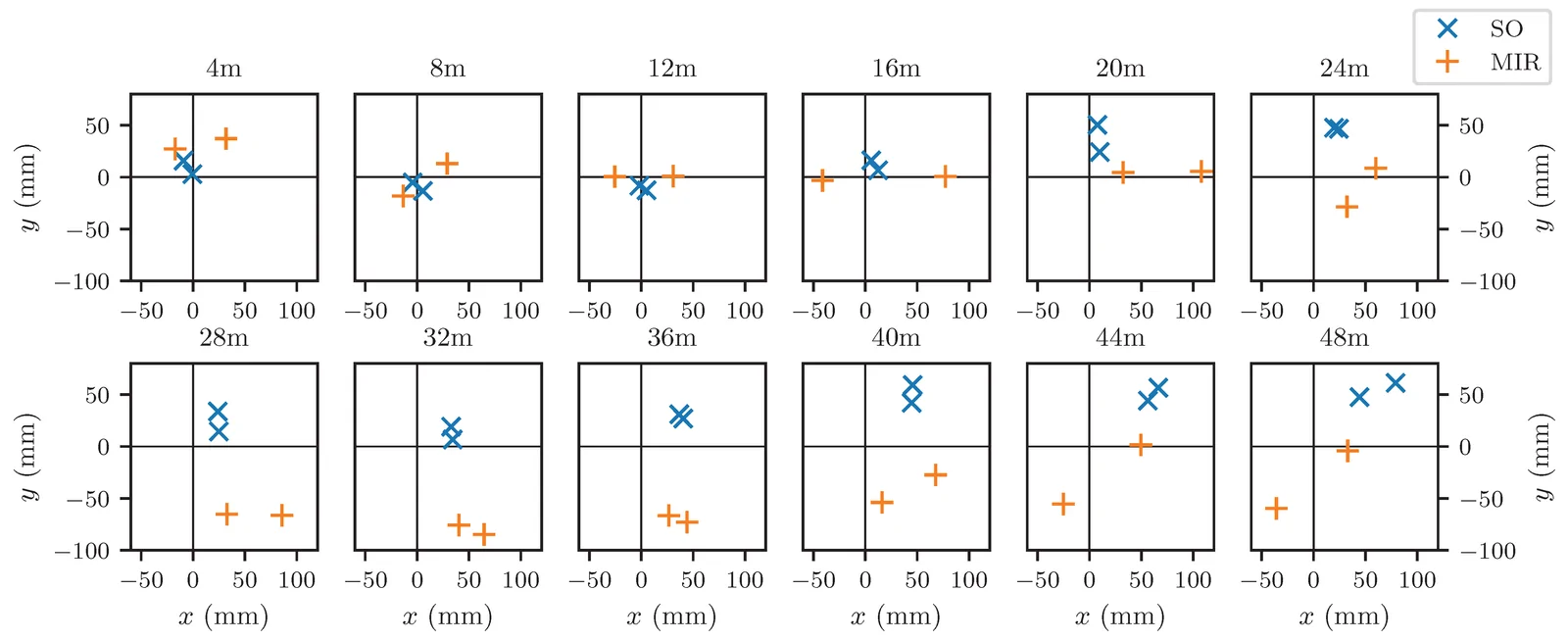
Position errors calculated from the laser tracker data at 4 m intervals around the 16×8 m path while the robot was stationary. The data show two runs with both the MIR’s SLAM (MIR) and the speckle odometry (SO).

**Figure 12:**
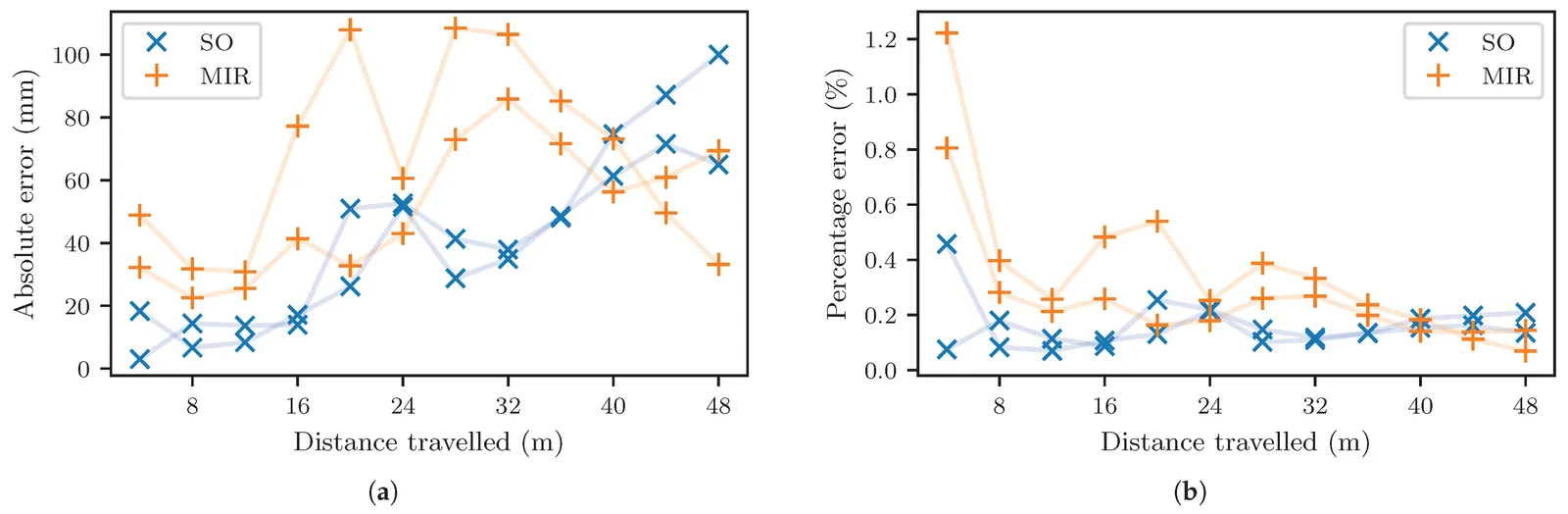
(**a**) Absolute magnitude of the position errors calculated from the laser tracker data at 4 m intervals around the 16×8 m path. (**b**) The same data as a percentage of distance travelled.

**Figure 13:**
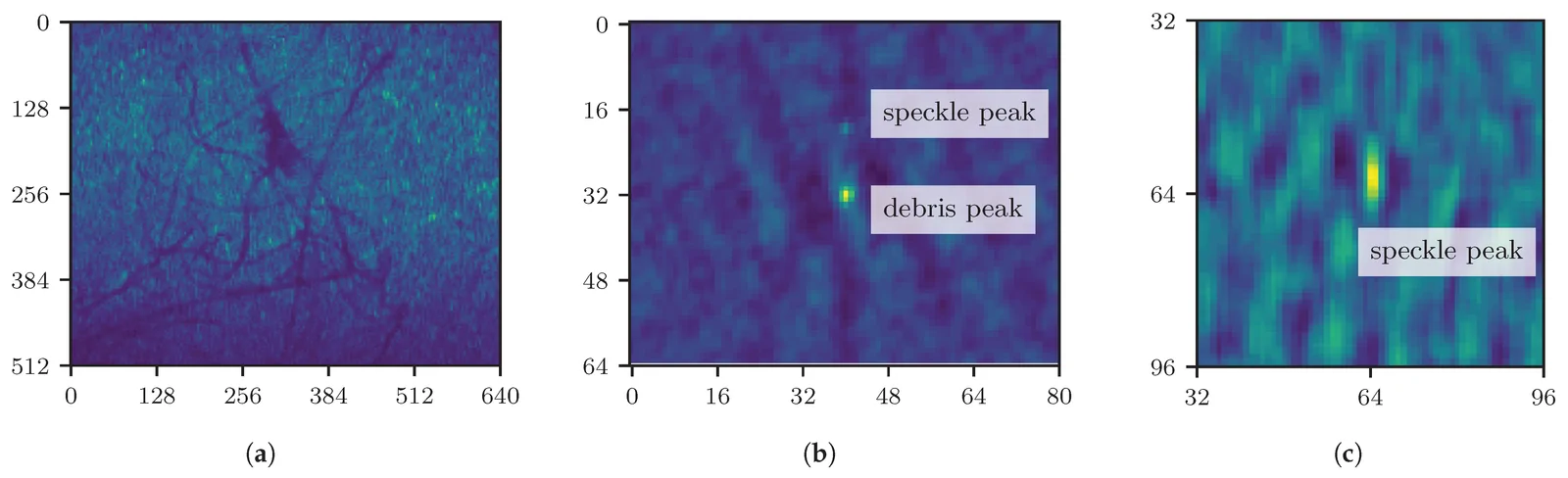
(**a**) Speckle image showing debris picked up by sensor. (**b**) Binned correlation image showing two peaks, one from the speckle patterns and one from the debris. (**c**) Windowed correlation image using manually-selected windows showing the true speckle peak.

**Table 1:** Odometry sensors using computer mice and/or laser speckle systems, a summary of the literature.

Description	Test Surface(s)	Test Path	Position Error
Two sensors with calibration, Bonarini et al. [8]	Carpet	4 × 4 m square (UMBmark test)	0.70%
Four sensors with calibration, Paijens et al. [12]	Concrete, PVC, wood	6.7 m arc	0.16%
Eight sensors with consistency check, Hu et al. [13]	Unknown, with/without transparent sheet	0.14 m radius circle	0.28–0.65%
Single sensor with external laser source, Nagai et al. [9]	Black and white paper, aluminium, carpet, artificial lawn, sand, stone	0.2 m line	0.20–1.60%
	Asphalt, paving	190 m arbitrary path	0.89%
Weighted sensor pair with external lenses, Ross et al. [14]	Asphalt	0.1 m line	1.94%
Eight sensors with external lenses, Dahmen and Mallot [15]	Patterned tile	0.45 m radius circle, 10 m arbitrary path	1.00%
Single afocal sensor plus sensor fusion, Yi et al. [16]	Carpet	1.5 × 2.0 m rectangle	0.62%
Two sensors plus sensor fusion, Tajti et al. [17]	Unknown	0.5 × 0.5 m square	1.00%
Single sensor plus sensor fusion, Liu et al. [18]	White paper	1.6 m line, 34 m random walk	0.60%
Custom speckle sensor, Francis et al. [20]	Martian soil analogue	1.12 m S-shape	0.35%
Custom speckle sensor with two cameras, Jo et al. [21]	Paper with checkerboard pattern, poster	0.01 m line	2.6%

## Data Availability

The original data presented in the study are openly available in the Cranfield Online Research Data Repository at https://doi.org/10.57996/cran.ceres-2873.
